# Computational and Experimental Optimization of Injection-Molded Compliant Constant-Torque Mechanisms in Polymeric Materials

**DOI:** 10.3390/polym17182505

**Published:** 2025-09-17

**Authors:** Tran Minh The Uyen, Hai Nguyen Le Dang, Van-Thuc Nguyen, Minh-Tai Le, Nguyen Van Son, Thanh Trung Do, Le Quang Linh, Vu Manh Hoang, Phi Hoang Minh, Pham Son Minh

**Affiliations:** Faculty of Mechanical Engineering, Ho Chi Minh City University of Technology and Education, Ho Chi Minh City 71307, Vietnam; uyentmt@hcmute.edu.vn (T.M.T.U.); hainld@hcmute.edu.vn (H.N.L.D.); nvthuc@hcmute.edu.vn (V.-T.N.); tailm@hcmute.edu.vn (M.-T.L.); sonnv@hcmute.edu.vn (N.V.S.); trungdt@hcmute.edu.vn (T.T.D.); 19143026@student.hcmute.edu.vn (L.Q.L.); 19144071@student.hcmute.edu.vn (V.M.H.); 19144156@student.hcmute.edu.vn (P.H.M.)

**Keywords:** ANN, experiment, CAE, constant-torque mechanisms (CTMs), injection molding

## Abstract

In this research, we explore the computational and experimental optimization of compliant constant-torque mechanisms (CTMs) fabricated via injection molding using polymeric materials. We investigate how geometric variations influence the torsional strength of CTMs through numerical simulation, experimental validation, and artificial neural network (ANN) modeling. Four different geometries with the same overall dimensions were designed and analyzed to quantify their mechanical performance. The results reveal that the geometric configuration significantly affected the torsional behavior of the CTMs, with circular cross-sections demonstrating superior strength. Moreover, the ANN model exhibited a high prediction accuracy and minimal relative errors, closely aligning with the experimental outcomes. Despite this, discrepancies between our numerical and experimental results suggest that further refinements in material modeling and manufacturing processes are warranted. In this paper, we emphasize the importance of integrating computational (CAE), artificial neural networks (ANNs) and experimental techniques for optimizing polymer-based CTMs. CAE simulations for Model 4 showed a constant-torque section from 23–44 degrees with 0.142 N·m torque, while experimental and ANN results indicated a longer range (20–45/46 degrees) with higher torque values (0.164 N·m). Experimental and ANN predictions for Model 4 showed an approximate 97% similarity. While these findings represent a foundational step, the characteristics of polymer CTMs suggest potential relevance for advancing applications in precision engineering, biomedical devices, and soft robotics, pending further application-specific validation.

## 1. Introduction

Compliant constant-torque mechanisms (CTMs) have become increasingly popular in various engineering applications due to their ability to provide a constant force or torque output while eliminating the need for traditional components such as springs, gears, and bearings (Frischknecht et al. [1]). These mechanisms rely on the elastic deformation of their structural elements to achieve the desired functionality, making their design and analysis crucial for ensuring reliable performance, as noted by Kolovsky et al. [2]. One important aspect of compliant mechanism design is the consideration of the structural elements’ geometric shape, as this can significantly impact the torsional strength and, consequently, the overall performance of the mechanism (Sydenham et al. [3]).

However, existing studies have mainly focused on metal-based or macro-scale compliant mechanisms. There is a lack of comprehensive studies that simultaneously combine computational simulations, experimental validation, and predictive modeling using artificial intelligence for polymer-based injection-molded CTMs. This study addresses this gap by investigating the influence of geometric variations on torsional strength through an integrated approach combining finite element analysis, experiments, and ANN modeling.

One approach to addressing these challenges is through the use of active structures, which involve the direct control of single parameters when there is a closed-form relationship between the response required and the control parameter. Building on this concept, researchers have explored the use of active tensegrity structures, where closely coupled strut and cable elements exhibit nonlinear behavior even for small movements of the telescopic struts. The control criterion for maintaining the upper surface slope in these structures has no closed-form relationship with strut movement, underscoring the need for a more comprehensive understanding of the relationship between geometric shape and torsional strength.

Another promising approach is the use of hinge-free and fully decoupled compliant mechanisms, which can solve output coupling and input coupling problems. These mechanisms are well suited for applications such as cell manipulations and electronic microscopes, where movement uncoupling and maximum desired output displacements are critical (Rong et al. [4]). Researchers have also explored the use of tensegrity robots, which are based on carbon struts and springs, and are actuated by vibrators, the frequency of which is automatically tuned by a trial-and-error learning algorithm.

The compliance of the springs and the tensegrity structure of these robots allow them to maintain their integrity when deformed and spring back into their initial form, suggesting that the geometric shape of the mechanism may play a crucial role in its torsional strength and overall performance.

The findings of previous studies have indicated that the geometric shape of the structural elements in a compliant constant-torque mechanism can have a significant impact on its torsional strength. Specifically, we found that circular cross-sections exhibited the highest torsional strength, followed by elliptical and rectangular cross-sections (Kounadis et al. [5]). This can be attributed to the inherent structural properties of these shapes, with a circular geometry providing a more uniform stress distribution under torsional loading, according to Zheng and Zeng et al. [6]. These findings have important implications for compliant constant-torque mechanism design. First, they highlight the need to carefully consider the geometric shape of the structural elements when designing such mechanisms to ensure optimal torsional strength and, ultimately, reliable performance. Second, the results suggest that a circular cross-section may be the most suitable choice for applications where torsional strength is a critical design consideration, as suggested by Xu et al. [7]. Additionally, the use of superelastic materials like NiTiNOL can further enhance the torsional capabilities of compliant mechanisms, as demonstrated by Liu et al. [8]. Recent research trends have focused on developing large-stroke mechanisms [9,10,11]. These studies have been documented well in various statistical analyses [12,13].

Compliant mechanisms are commonly manufactured using cutting machining methods. However, this approach presents several limitations. Firstly, machining thin regions of mechanisms is difficult due to the associated low stiffness and high deformation risk [14]. Secondly, the large amount of excess material is wasteful, making it unsuitable for mass production [15]. Thirdly, the machining process is time-consuming, especially for complex components, leading to increased production costs [16]. Fourthly, residual stresses are generated during machining, which can reduce the lifespan of the compliant mechanism [17]. Finally, conventional machining is not ideal for large-scale manufacturing, as it is expensive and requires skilled labor [18].

In this study, we propose fabricating compliant mechanisms from plastic using the injection molding method, which offers significant benefits. First, injection molding enables high-speed mass production, significantly reducing manufacturing costs [19,20]. In addition, this method allows for the creation of highly precise components, suitable for complex designs [21,22]. Furthermore, since there is no cutting process, the products are less affected by residual stresses, enhancing their overall durability [23,24]. Injection molding also achieves a high material efficiency, minimizing waste and being more environmentally friendly compared to traditional machining [25,26]. Lastly, plastics possess excellent elasticity properties, enhancing the performance of compliant mechanisms and optimizing their load-bearing and elastic deformation capabilities [27,28].

However, the injection molding process also presents several challenges. Controlling the temperature distribution in the mold is crucial for product quality, as poor management can lead to dimensional inaccuracies or surface defects [29]. Additionally, shrinkage and warpage during cooling can cause substantial geometric errors, particularly in high-precision components [30]. Another challenge is selecting an appropriate plastic material to ensure mechanical strength and elasticity, which requires thorough research on polymer properties [31]. Moreover, process parameters such as the injection pressure, injection speed, and holding time need to be optimized to achieve the best quality possible [32]. These challenges underline the need for further research to improve the performance and precision of compliant mechanisms produced via injection molding.

In this research, we explore the influence of four geometric shapes on the torsional strength of compliant constant-torque mechanisms fabricated using injection molding. The selection of shapes was random and not based on specific parameters. Polypropylen (PP) was chosen due to its availability, low cost. Our goal is to provide actionable insights that inform the design and optimization of these mechanisms. To achieve this, we developed a suite of numerical models to evaluate the torsional behavior of compliant constant-torque mechanisms with varying geometric shapes. By integrating numerical simulation, experimental validation, and artificial neural network (ANN) as novel approaches, we have successfully bridge critical gaps in the knowledge in this field. The models were subjected to a range of torsional loading conditions, and the resulting deformation and stress distributions were analyzed to quantify and compare the torsional strength of each geometric configuration. The research aims to understand how variations in geometric design influence the torsional strength of Constant-Torque Mechanism (CTM) models.

## 2. Materials and Methods

### 2.1. Geometric Shape Selection

In this research, four proposed designs—Model 1, Model 2, Model 3, and Model 4—have share certain dimensions, including an outer rim diameter of 100 mm, a thickness of 3 mm, and a compliant beam width of 1 mm. The concepts for these models—circular, were derived from the methodologies discussed in [11,12,13]. The models were made of polypropylene (PP), which has a yield strength of 34.6 MPa, Young’s modulus of 1.461 GPa, and Poisson’s ratio of 0.4087.

**Figure 1:**
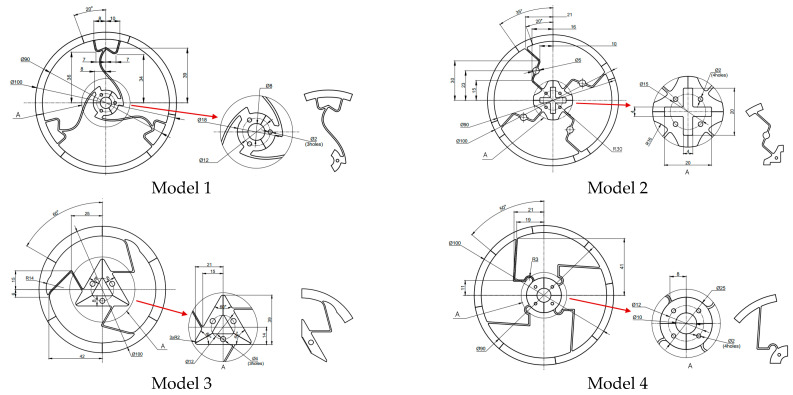
Four models designed using 3D software.

In Figure 1, from left to right, four models of compliant mechanisms are shown. Models 1 and 3 have three compliant beams, while models 2 and 4 have four. Model 1 was designed with a curved beam, while the others were designed with straight beams. These models were produced using an injection molding machine. Each model was then divided into four pieces.

### 2.2. Part Manufacturing

**Figure 2:**
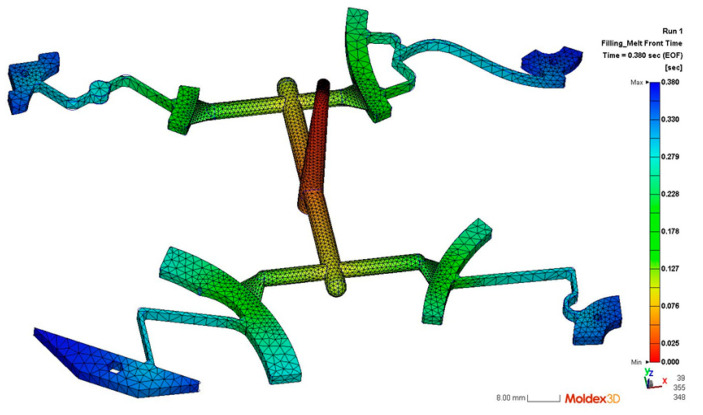
Injection products.

We employed Moldex3D software (2025) to simulate the injection molding process itself, aiding in visualizing material flow (Figure 2) and selecting feasible initial processing parameters, rather than directly optimizing the final mechanical torque performance in this step. And then four injection moldings sets were machining by CNC machine.

In this research, the products were manufactured using a HAITIAN MA1200III injection molding machine, which had a clamping force of 12,000 kN and an injection volume of up to 4500 cm^3^. One shot can produce up to 4 different pieces, as shown in Figure 2; this process was repeated for the remaining three models. Polypropylene (PP) [33] was selected as the CTM material due to its excellent fatigue strength, making it a preferred choice for cyclic load-bearing hinge couplings.

Here, 5 input parameters—referred to as injection pressure (45 MPa, 46 MPa, 47 MPa, 48 Mpa, and 49 MPa); holding pressure (30 MPa, 32 MPa, 34 MPa, 36 MPa, and 38 MPa); melting temperature (220 °C, 222 °C, 224 °C, 226 °C, and 228 °C); mold temperature (30 °C, 35 °C, 40 °C, 45 °C, and 50 °C); and holding time (1 s, 1.5 s, 2 s, 2.5 s, and 3 s)—all have 5 levels. The input parameters and the levels were chosen based on our previous experience. As shown in Table 1, Taguchi L25 (5^6^) for five factors and five levels was applied for four models to find the optimal value that produces the best torque value.

### 2.3. Torsion Simulation

In order to generate the relationship between the reaction torque and applied displacement, a static analysis was carried out with ANSYS 2024 software to simulate the torque change along with the variation in input stroke. The CAE simulation assumed the material to be homogeneous and isotropic, and the analysis was conducted within the elastic/small plastic deformation range relevant to the mechanism’s function. The following initial conditions were selected: number of steps—75; initial time—0.5 s; minimum time step—0.5 s; maximum time step—1 s; rotation—Z.

In this study, we assumed the material to be homogeneous and isotropic, with the stress remaining within the material’s elastic limit. A boundary condition was applied with a fixed support at the center. The same force was applied to each design, and the analysis aimed to identify the designs exhibiting the best maximum and minimum stress responses.

**Figure 3:**
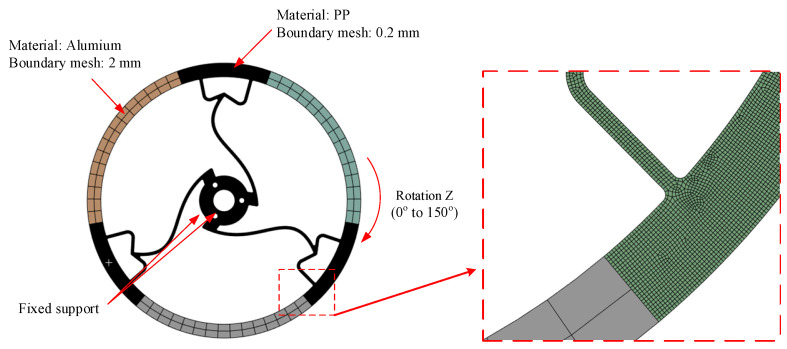
Meshing and boundary conditions of Model 1.

As shown in Figure 3, the model setup had Polypropylene (PP) as the material, with boundary mesh sizes specified as 2 mm. A fixed support was applied to a designated point or section, while the component underwent rotation along the *Z*-axis within a range of 0° to 150°.

**Figure 4:**
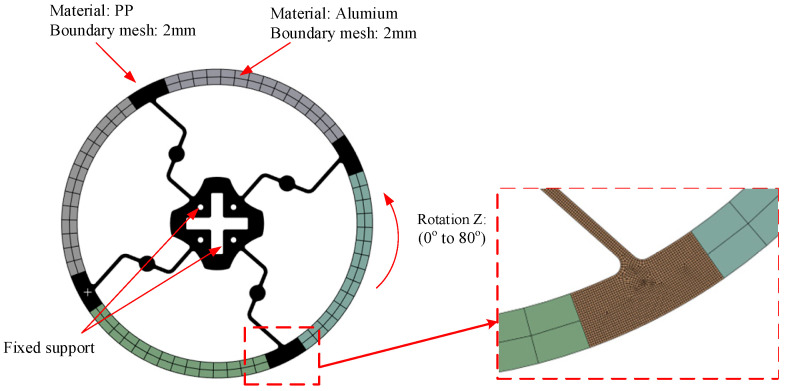
Meshing and boundary conditions of Model 2.

As shown in Figure 4, the material and condition setup was the same as that shown Figure 3, within a range of 0° to 80°.

**Figure 5:**
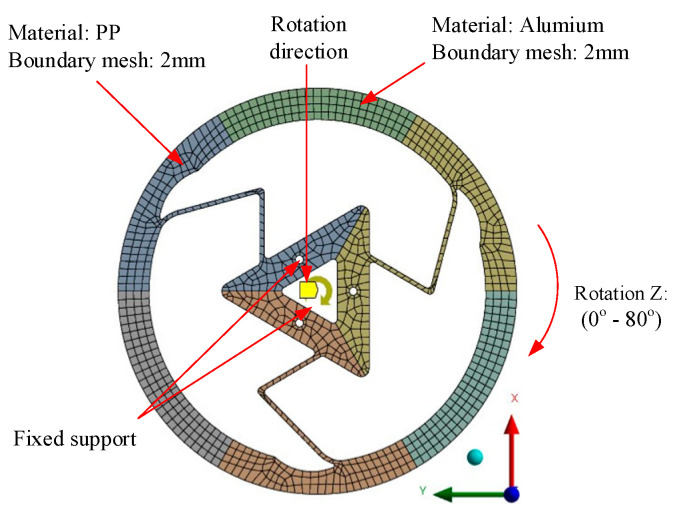
Meshing and boundary conditions of Model 3.

As shown in Figure 5, the material and condition setup was the same as that shown previously, within a range of 0° to 80°.

**Figure 6:**
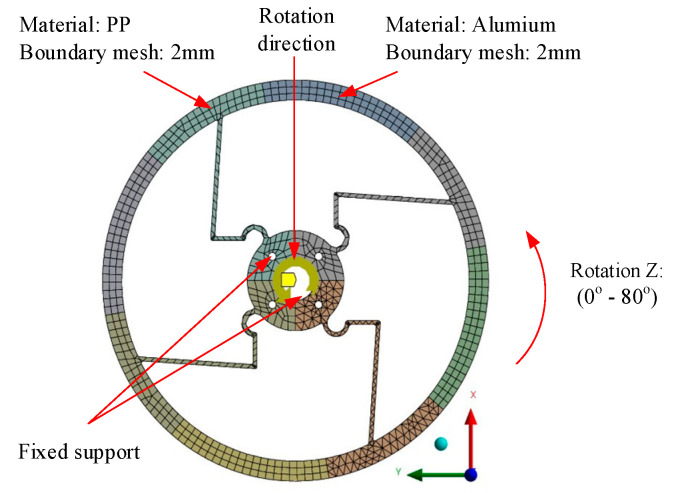
Meshing and boundary conditions of Model 4.

As shown in Figure 6, the material and condition setup was same as that shown previously, within a range of 0° to 80°.

### 2.4. Experimental Validation

The system consisted of three units: the machine, electric cabinet, and laptop. The torsion test machine applied a controlled 0–300 Nm load via a 40 W AC synchronous motor, transmitting torque through a 1:2 timing belt pulley. Operating at 10 RPM, the motor ensured stable moments with minimal slippage. A hand wheel enabled manual shaft rotation. Torque amplification was achieved via a 1:50 planetary gearbox. A belt tension mechanism and a 1000-pulse encoder (1:1 belt drive) measured rotation angles. The data acquisition unit, equipped with three-jaw chucks for precise specimen alignment, integrated a torque sensor, with the error margin of ±0.005 N·m for real-time data collection.

**Figure 7:**
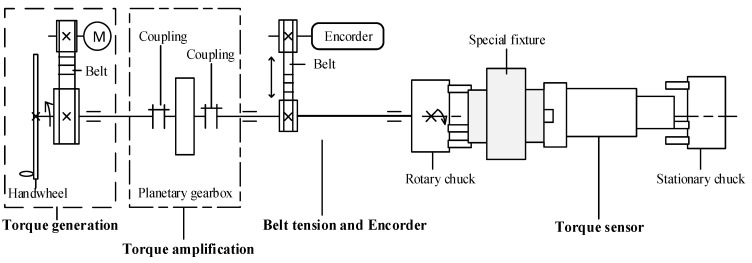
Kinematic diagram of the torsion test machine.

Figure 7 illustrates the kinematic diagram of the torsion test machine. The input of the system is the rotation and torque of the motor. The output is the measured torque and the rotary angle.

Next, the kinematic balance equation of the machine is presented as follows:

One revolution of motor × $\frac{1}{2}$ × $\frac{1}{40}$ = Angle. With this motor at 10 rpm, the recorder angle of the encoder is 45 degrees per minute, which is the twist rate of the machine.

Here, the power of the machine is lost through the bearings, toothed belts, couplings, and gearbox.

The angle of twist, θ [34] can be calculated for a linear elastic material according to the following equation:

(1) $$\theta =\frac{TL}{JG}$$

where *T* denotes the applied torque (N·m).

J is the second polar moment of the area of the cross-section and depends on the cross-section of the test specimen. The following are two popular cross-sections:

G is the modulus of rigidity or shear modulus (Pa), which can be calculated based on Equation (2):

(2) $$G=\frac{TL}{\theta J}$$

Equation (1) shows the direct proportion relation of the torque (T), length (L) with angle of twist (∅), the inverse ration of polar moment of the area (J), and the modulus of rigidity (G).

**Figure 8:**
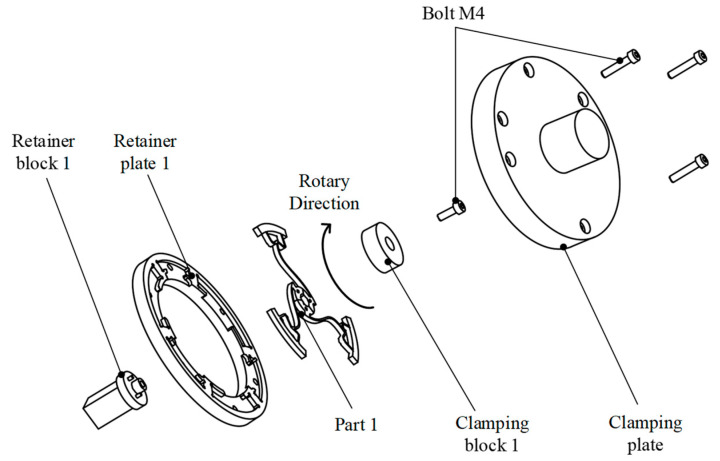
Special component designed to clamp Model 1.

Figure 8 illustrates the custom-designed jig used to securely hold Model 1 in place within the machine. There are four distinct jigs for the four models. Note: Axis labels and scale bars are included in the updated version to enhance clarity.

**Figure 9:**
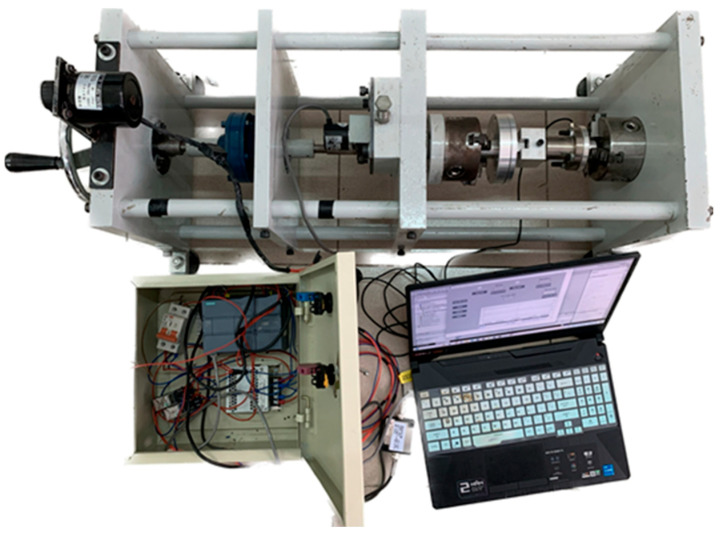
The torsion machine in working.

Figure 9 shows the actual torsion test machine at the top, electric cabinet on the left, and the control laptop on the right. From right to left, the machine comprised a torque generation mechanism, torque amplifier, belt tension and encoder, fixture compartment inside the machine frame. One end of the digital torque adapter mounted directly to the stationary chuck was used to measure the torque of the test specimen. It was attached to a stationary chuck through an intermediate part designed to fit the input and output of the sensor. The adapter acquired torque from the sample, allowing for the observation and recording of real-time torque data.

### 2.5. ANN Toolbox with MATLAB 2018 Software

The artificial neural network (ANN) model involves computations and mathematics, which simulate human brain processes. When compared to other models, the ANN model—more precisely, a multilayer neural perceptron, or MLP—performed better in terms of the Minimum Root Mean Square Error (RMSE) (0.50), AARD (100.87), and Maximum Coefficient of Determination R2 (0.90), demonstrating high accuracy and low relative error [12,35]. Many researchers have employed ANNs in previous studies [36,37]. The network architecture consists of an input layer, two hidden layers (10 and 8 neurons), and an output layer. The injection pressure, molding pressure, plastic temperature, mold temperature, molding duration, rotation angle, and the target torque are all contained within the input layer. The output layer contains the predicted torque.

## 3. Results and Discussion

### 3.1. Numerical Simulation Results

The CAE simulations using ANSYS provide insights into the stress distribution and torque-rotation behavior based on the idealized model. The aim was to simulate realistic operating conditions by applying angular displacements that resemble actual mechanical loading, sufficient to capture the constant-torque region characteristic of each design. These angles were determined through preliminary simulations and literature references to ensure that each geometry reached its typical operational deformation range, including the onset of significant resistance increase, without exceeding material failure limits within the simulation.

**Figure 10:**
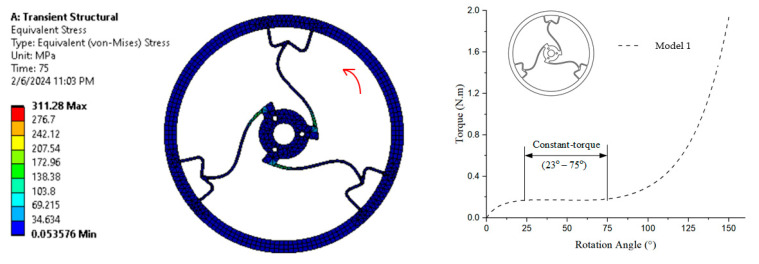
von Mises stress and torque–rotation angle curve of Model 1.

In Figure 10, the simulation result on the left shows the equivalent (von Mises) stress distribution, with stress levels ranging from 0.05376 MPa (blue) to 311.28 MPa (red) after a transient structural analysis conducted over 75 time steps, as depicted in the color map. The constant torque region exhibits torque values ranging from approximately 0.164 N·m to 0.167 N·m, corresponding to a rotation angle between 23° and 75°, which satisfies the definition Constant torque.

**Figure 11:**
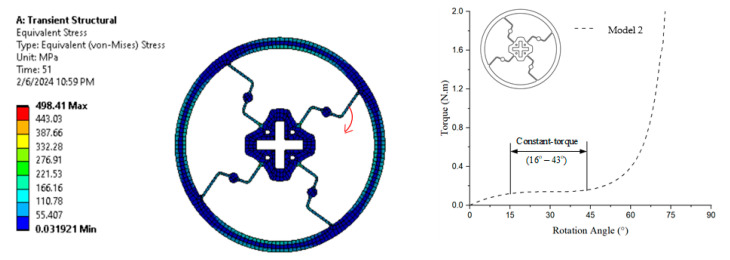
von Mises stress and torque–rotation angle curve of Model 2.

The numerical simulation in Figure 11 shows the equivalent (von Mises) stress distribution, with stress levels ranging from 0.03192 MPa (blue) to 498.41 MPa (red) after a transient structural analysis conducted over 51 time steps. The graph illustrates that the constant-torque region exhibits torque values ranging from approximately 0.137 N·m to 0.140 N·m, corresponding to a rotation angle of approximately 16° to 43°.

**Figure 12:**
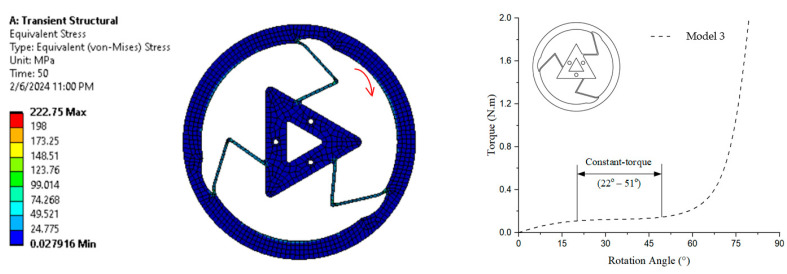
von Mises stress and torque–rotation angle curve of Model 3.

The numerical simulation in Figure 12 shows the equivalent (von Mises) stress distribution, with stress levels ranging from 0.0279 MPa (blue) to 222.75 MPa (red) after a transient structural analysis conducted over 50 time steps. The curve on the right demonstrates that the constant-torque region is characterized by torque values ranging from approximately 0.127 N·m to 0.130 N·m, with a rotation angle spanning from 22° to 51°.

**Figure 13:**
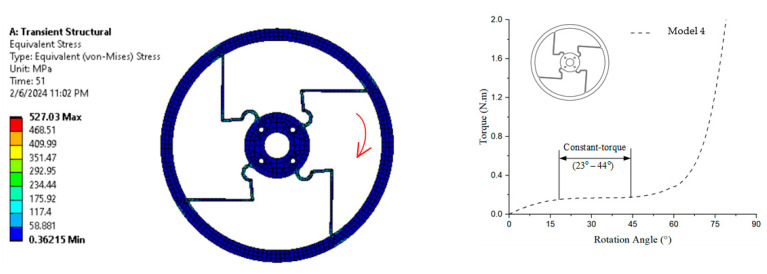
von Mises stress and torque–rotation angle curve of Model 4.

The simulation result in Figure 13 shows the equivalent (von Mises) stress distribution, with stress levels ranging from 0.36215 MPa (blue) to 527.03 MPa (red) after a transient structural analysis conducted over 51 time steps. It can be observed that the constant-torque region has torque values of approximately 0.142 N·m to 0.144 N·m, with a rotation angle ranging from about 23° to 44°.

**Figure 14:**
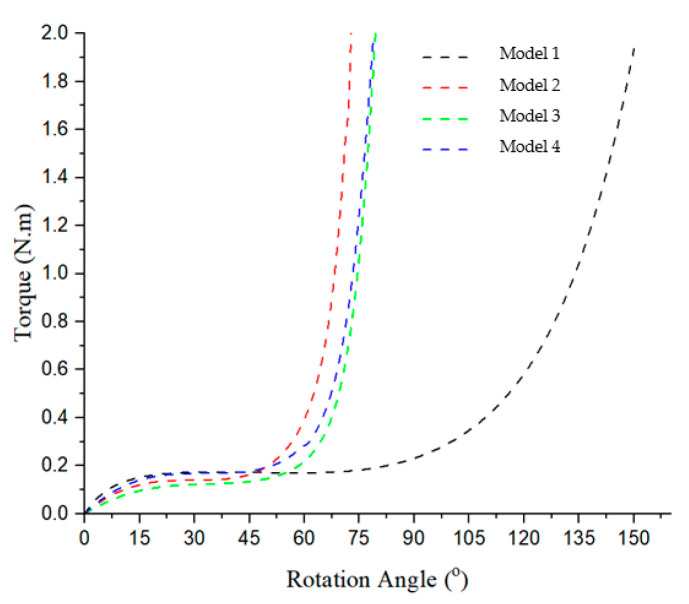
Torque–rotation curve of four models.

From Figure 14, it is apparent that all of the models’ curves shows a trend similar to that of the red curve in Figure 7 presented in [12]. Model 1 exhibits the highest torque value and rotation angle, ranging from approximately 23° to 75°, with a torque value reaching 0.165 N·m. In contrast, Model 2 displays the smallest torque value and rotation angle, spanning from about 16° to 43°, with a torque value of approximately 0.139 N·m. Model 3 and Model 4 display the same trend.

### 3.2. Experiment Results

The accuracy of the torsion test machine is crucial to accurately determine the torsional strength and behavior of the materials. Therefore, it is important to perform a function test on the torsion test machine to ensure its proper functioning and accuracy. Various parameters and functionalities of the machine are evaluated during the function test of a torsion test machine. These include verifying the load capacity, testing the rigidity of the machine structure, checking the precision of the torque measurements, assessing the alignment of the specimen grips, and examining the stability and repeatability of the test results. By performing a thorough function test on the torsion test machine, potential issues or inaccuracies can be identified and addressed, ensuring reliable and accurate testing results for evaluating materials’ mechanical properties.

Each model has 25 cases. From the results obtained using the torsion machine, a set of parameters for each sample that gave the longest constant torque was chosen.

**Figure 15:**
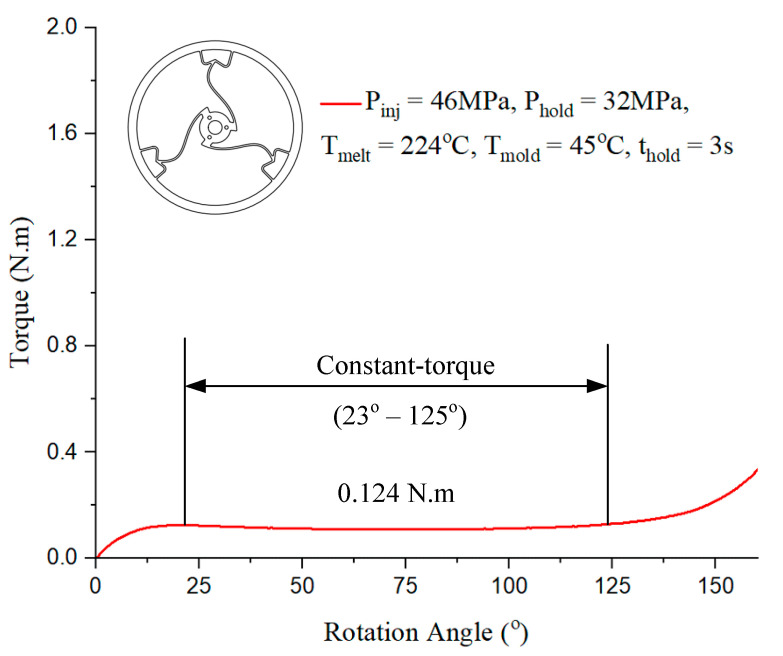
Torque–rotation curve from experiment with Model 1.

In Figure 15, the constant-torque region exhibits torque values ranging from approximately 0.124 N·m to 0.124 N·m, corresponding to a rotation angle between 23° and 125°. For Model 1, case 7 presented in Table 1 with the injection parameter in Figure 15 could produce the best output.

**Figure 16:**
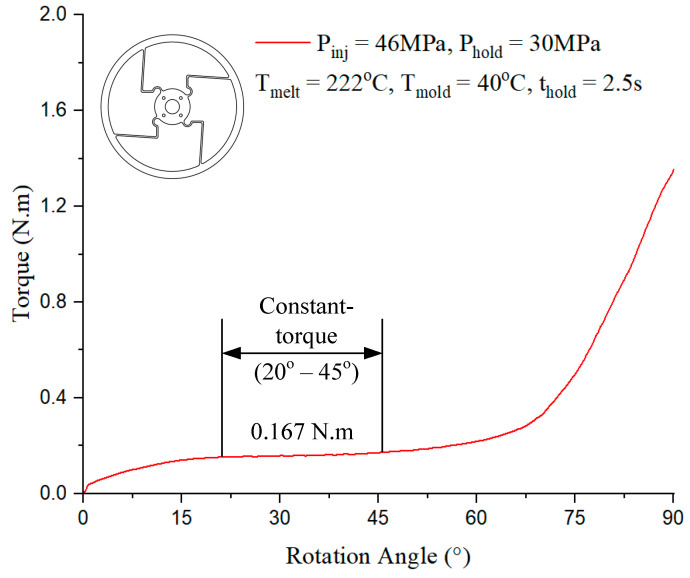
Torque–rotation curve from experiment with Model 2.

In Figure 16, the constant-torque region exhibits torque values ranging from approximately 0.167 N·m to 0.167 N·m, corresponding to a rotation angle between 20° and 45°. For Model 2, case 6 presented in Table 1 with the injection parameter in Figure 16 could produce the best output.

**Figure 17:**
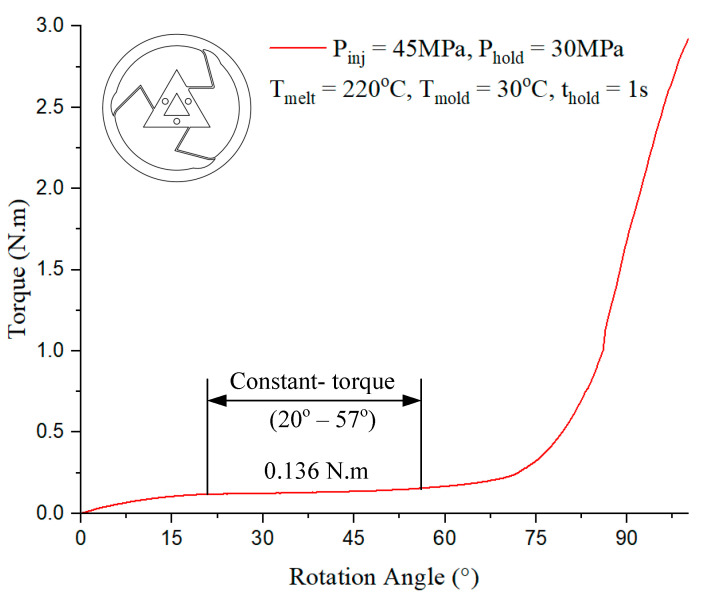
Torque–rotation curve from experiment with Model 3.

In Figure 17, the constant-torque region exhibits torque values ranging from approximately 0.136 N·m, corresponding to a rotation angle between 20° and 75°. For Model 3, case 1 presented in Table 1 with the injection parameter in Figure 17 could produce the best output.

**Figure 18:**
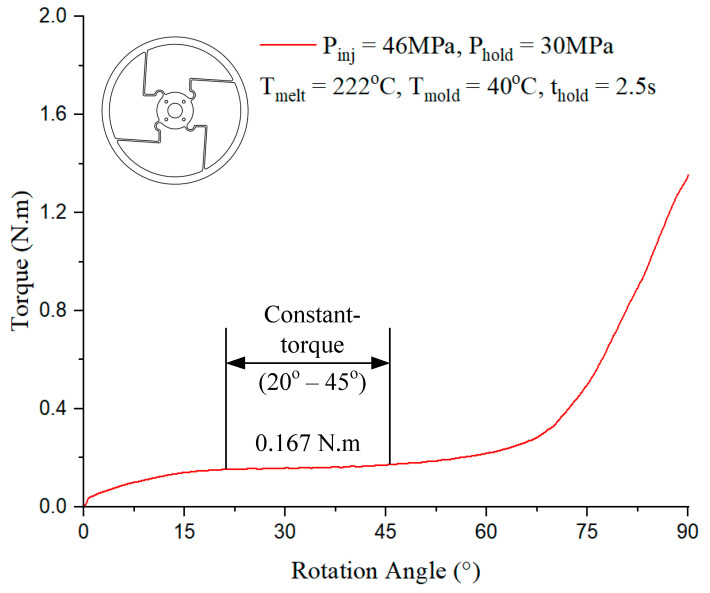
Torque–rotation curve from experiment with Model 4.

In Figure 18, the constant-torque region exhibits torque values ranging from approximately 0.167 N·m, corresponding to a rotation angle between 20° and 55°. For Model 4, case 6 presented in Table 1 with the injection parameter in Figure 18 could produce the best output.

**Figure 19:**
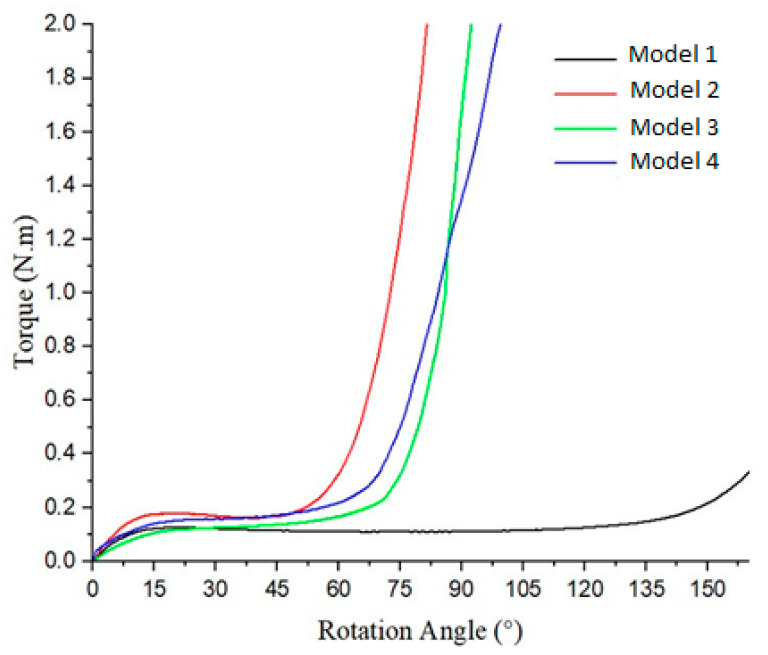
Torque–rotation curve from experiments with four models.

The four curves illustrate the behavior of the torques derived from testing the different models (Figure 19). A sharp increase in the torque beyond a specific angle indicates the point where the parts begin to resist rotation more significantly. Models 1 and 2 exhibit relatively low resistance, with Model 2 displaying a slightly higher torque than that in Model 1. In contrast, Models 3 and 4 show a more pronounced and continuous increase in torque, with Model 4 demonstrating the highest level of resistance.

### 3.3. Prediction Output of ANN

In this study, four artificial neural network (ANN) models were developed to predict the bending moment of compliant mechanism designs based on six process parameters: injection pressure, packing pressure, melt temperature, mold temperature, packing time, and rotation angle. The network architecture consisted of a feed-forward multilayer perceptron with three hidden layers (128, 64, and 32 neurons, respectively) and a single output neuron representing the predicted moment. The ReLU activation function was adopted in all hidden layers, and the Adam optimizer was employed with an adaptive learning rate initialized at 0.001. Early stopping with a tolerance of 1 × 10^−4^ was applied to avoid unnecessary training epochs. The training process was conducted for up to 2000 iterations, and convergence was generally achieved after approximately 1000 iterations, where the training error dropped below 0.0008.

The dataset used for training and evaluation comprised 22,975 samples after data cleaning. These data were randomly divided into three subsets: 60% for training, 20% for validation, and 20% for testing. In addition, a 5-fold cross-validation procedure was conducted to further ensure the robustness of the models.

**Figure 20:**
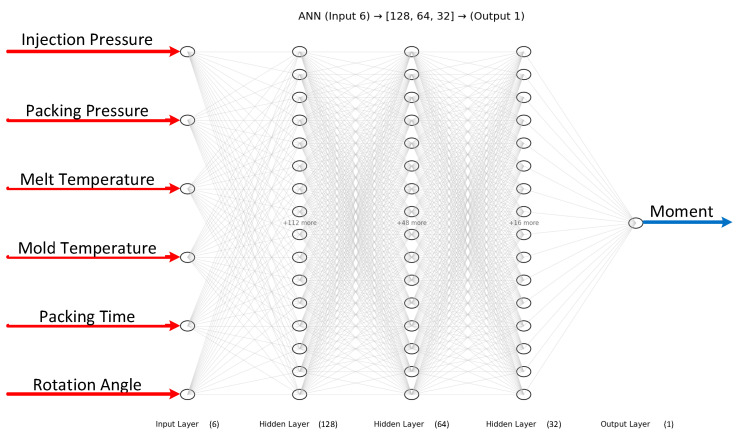
ANN structure for predicting 4 models.

**Figure 21:**
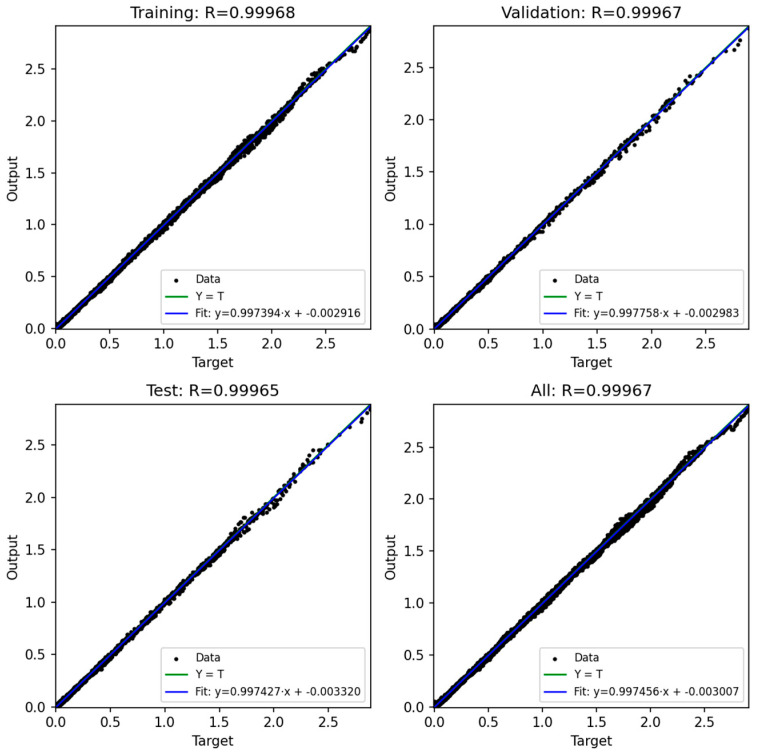
ANN result for Model 1.

**Figure 22:**
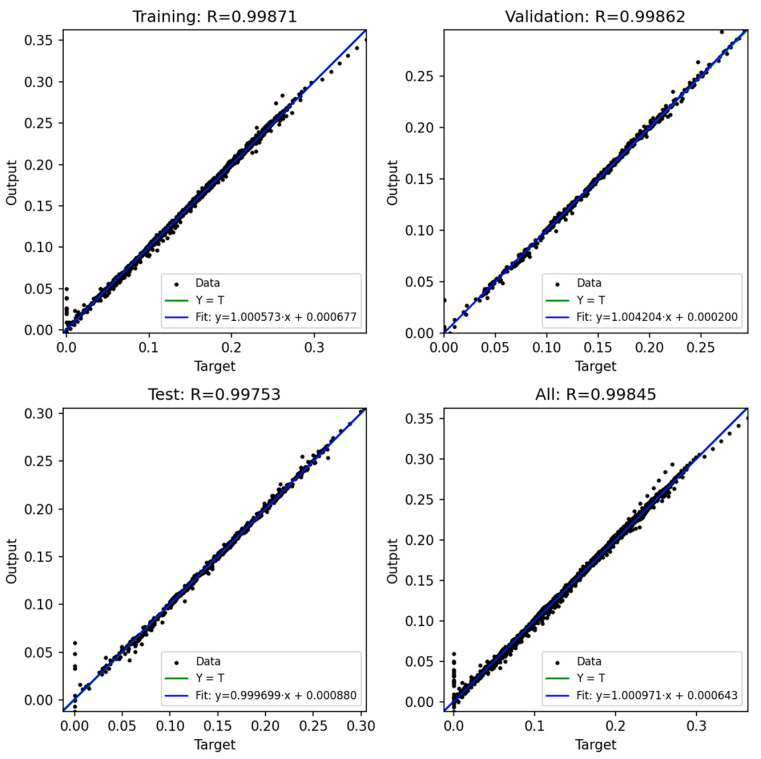
ANN result for Model 2.

**Figure 23:**
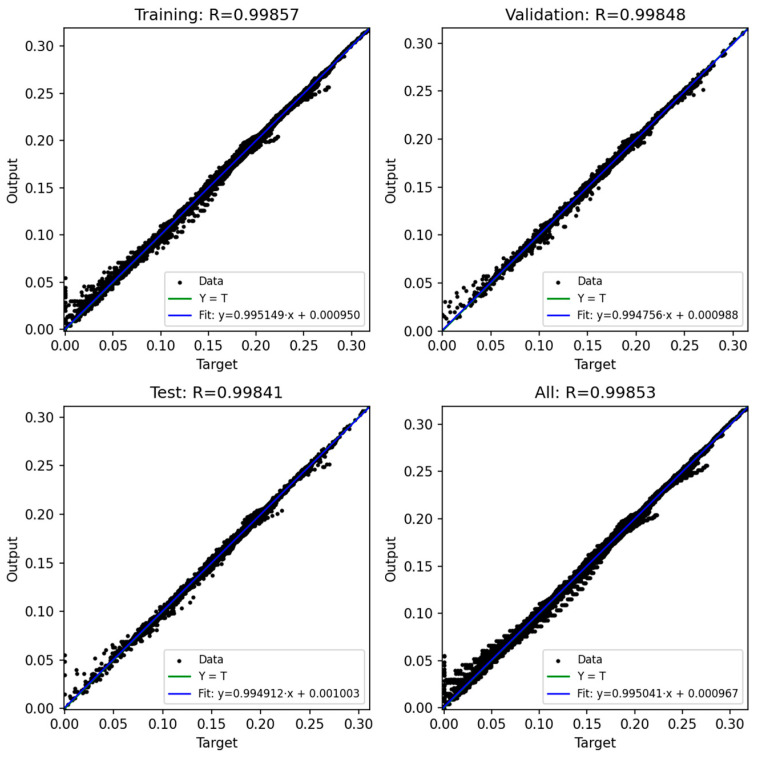
ANN result for Model 3.

**Figure 24:**
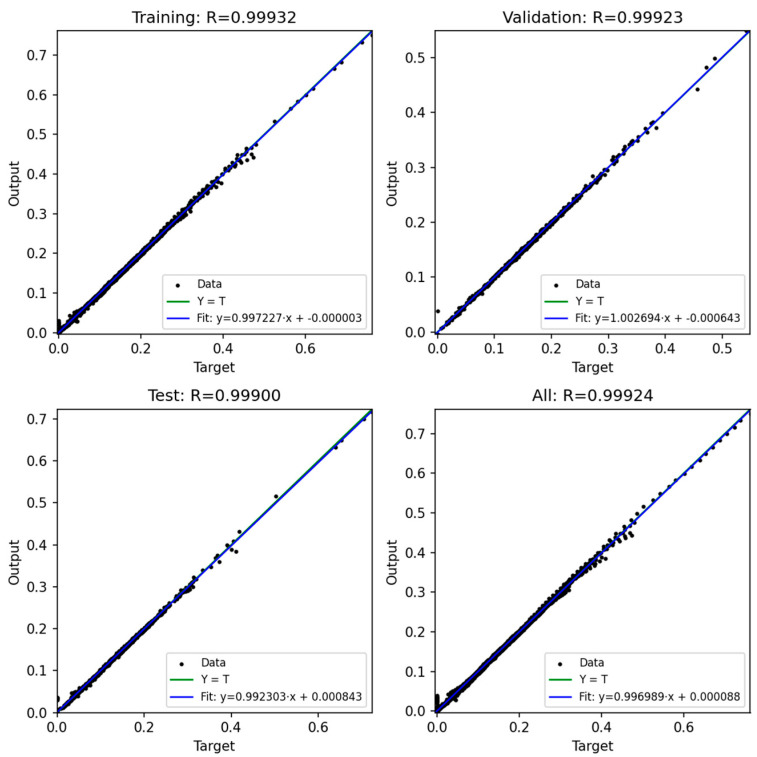
ANN result for Model 4.

Figure 20, Figure 21, Figure 22, Figure 23 and Figure 24 show the scatter plots of predicted versus actual outputs for the training, validation, test, and combined datasets. In all cases, the data points are closely aligned with the 45-degree line, confirming excellent agreement between predictions and targets. The quantitative performance of the four ANN models is summarized in Table 2. For Design 1, the coefficient of determination R^2^ reached 0.9993 across all datasets, with MAE = 0.0081 and RMSE = 0.0118 on the training set. Design 2 achieved R^2^ ≈ 0.9972 (train), 0.9970 (validation), and 0.9948 (test). Design 3 obtained R^2^ ≈ 0.9971 (train), 0.9969 (validation), and 0.9968 (test). Design 4 achieved R^2^ ≈ 0.9986 (train), 0.9984 (validation), and 0.9979 (test). These results indicate consistently high prediction accuracy.

Importantly, the overfitting checks (ΔR^2^ gaps and error ratios) demonstrate that the models generalize well. For all four designs, the ΔR^2^ between training and validation was below 0.0003, and the ΔR^2^ between training and test was below 0.0024. Similarly, the MAE and RMSE ratios between validation/test and training sets ranged mostly between 0.99 and 1.10, with only Design 2 showing slightly higher values (MAE ratio Test/Train ≈ 1.14, RMSE ratio Test/Train ≈ 1.38). These ratios close to unity confirm that the prediction errors on unseen data are comparable to those on training data, thereby proving that the networks are not overfitted.

Furthermore, the 5-fold cross-validation R^2^ values averaged between 0.9948 and 0.9994 with small standard deviations, further reinforcing the robustness of the models. Overall, the developed ANN architectures exhibited strong predictive capability and generalization across all datasets, providing reliable tools for modeling the mechanical response of compliant mechanisms.

**Figure 25:**
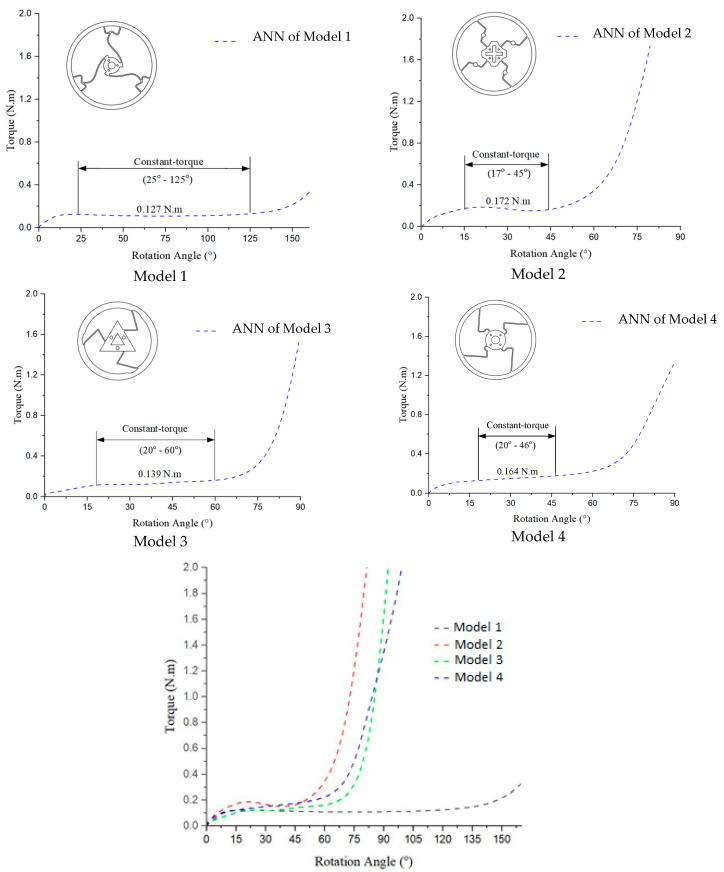
Torque–rotation curves from ANNs of 4 models.

In Figure 25, Model 1 exhibits the lowest torque output with a smooth, gradual increase across all of the rotation angles and the longest constant torque. Model 2 produces a higher torque than that with Model 1, with a slightly steeper increase. Model 3 generates the highest torque, especially at larger rotation angles, showing a rapid rise. Model 4’s performance falls between that of Models 2 and 3, featuring a smoother yet steeper torque increase than that with Models 1 and 2. Overall, all of the models demonstrate a nonlinear torque–angle relationship, indicating variations in design or mechanical characteristics that influence the torque behavior.

### 3.4. Comparison of Simulation, Experiment, and ANN Results

**Figure 26:**
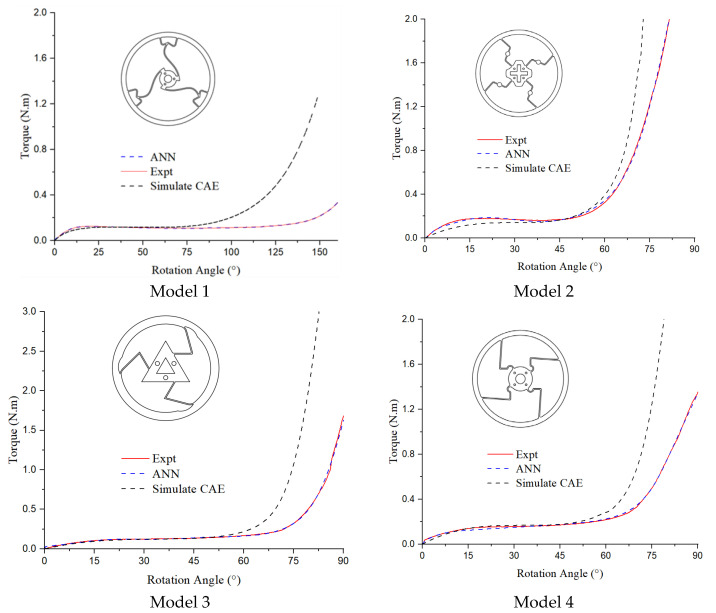
Torque–rotation curve of experiment, ANN, and CAE of four models.

In Figure 26, all four curves show that the results of the experiment and the predictions generated by the artificial neural network (ANN) exhibit an approximate similarity of 98%. The torque values are smaller than those derived from the numerical simulation, as the ANN’s predictions are derived from experimental data.

Table 3 shows that the range of constant-torque angles suggests that CAE typically produces narrower angle ranges compared to that produced by the EXP and ANN methods, which indicate more variability in their results. The constant-torque values seem to exhibit moderate variation, with EXP and ANN methods generally producing slightly higher torque values compared to that produced by CAE in most models. The differences across the methods could point to the varying effectiveness of the methods in predicting or measuring the physical parameters in different models.

The primary cause of this can be partially attributed to the disparity in the mechanical characteristics between the materials in the CAE and the external simulations, as well as environmental variables like humidity and temperature. Varied torque values are usually produced by products with varied designs.

## 4. Conclusions

The experimental torque values obtained in this study (0.124–0.167 N·m) are in good agreement with previous research on compliant constant-torque mechanisms. For instance, Phan et al. [9] reported torque levels of approximately 0.12–0.15 N·m for large-stroke compliant CTMs, while Bai et al. [10] achieved higher torque values through axial force release strategies. Compared with these approaches, the injection-molded CTMs developed in our work provide a simpler and more cost-effective solution in case of mass production while maintaining comparable torque performance.

In conclusion, the design and analysis of compliant constant-torque mechanisms (CTMs) play a crucial role in ensuring their reliable performance in various engineering applications. The main contributions and findings can be summarized as follows:

The geometric shape of structural elements is a significant factor impacting the torsional strength and overall performance of compliant mechanisms. Previous studies have indicated that circular cross-sections exhibit the highest torsional strength, followed by elliptical and rectangular cross-sections, highlighting the importance of geometric considerations in design. We explored the influence of geometric shapes on the torsional strength of compliant constant-torque mechanisms fabricated using injection molding, employing a comprehensive approach that integrated numerical models, experimental validation, and artificial neural network (ANN) techniques.

Remarkably, the ANN model displayed a high accuracy and low relative error, with its predictions closely aligning with those of the experimental results, providing valuable insights for future design and optimization.

Discrepancies between the simulation and experimental data were observed, potentially arising from variations in the material’s characteristics and environmental factors.

In conclusion, the findings emphasize that the geometry of structures must be considered seriously during the process of designing compliant constant-torque mechanisms in order to achieve an optimum torsional strength enabling the mechanisms to perform well under various design and environmental conditions.

## Figures and Tables

**Table 1:** Taguchi 25 cases.

	Parameters	$P_{inj}$ (MPa)	$P_{hold}$ (MPa)	$T_{melt}$ (°C)	$T_{mold}$ (°C)	$t_{hold}$ (s)
Case						
1		45	30	220	30	1
2		45	32	222	35	1.5
3		45	34	224	40	2
4		45	36	226	45	2.5
5		45	38	228	50	3
6		46	30	222	40	2.5
7		46	32	224	45	3
8		46	34	226	50	1
9		46	36	228	30	1.5
10		46	38	220	35	2
11		47	30	224	50	1.5
12		47	32	226	30	2
13		47	34	228	35	2.5
14		47	36	220	40	3
15		47	38	222	45	1
16		48	30	226	35	3
17		48	32	228	40	1
18		48	34	220	45	1.5
19		48	36	222	50	2
20		48	38	224	30	2.5
21		49	30	228	45	2
22		49	32	220	50	2.5
23		49	34	222	30	3
24		49	36	224	35	1
25		49	38	226	40	1.5

**Table 2:** Summary of evaluation for ANN models.

Metric	Design 1	Design 2	Design 3	Design 4
R^2^ (Coefficient of Determination—Train)	0.999279	0.997231	0.997123	0.998617
R^2^ (Validation)	0.999265	0.996996	0.996947	0.998425
R^2^ (Test)	0.999205	0.994841	0.996797	0.997947
R^2^ (All data)	0.999262	0.996713	0.997032	0.998452
MAE (Mean Absolute Error—Train)	0.008102602	0.001831427	0.001838897	0.001833411
MAE (Validation)	0.008295266	0.001986561	0.001894827	0.001817684
MAE (Test)	0.008519571	0.002090511	0.001878853	0.002026461
RMSE (Root Mean Square Error—Train)	0.011786869	0.003054946	0.002638221	0.002806038
RMSE (Validation)	0.011894471	0.003247533	0.002678101	0.002785516
RMSE (Test)	0.012471236	0.004225678	0.002728104	0.003432525
Cross-Validation R^2^ (mean ± std)	0.999358 ± 0.000054	0.994762 ± 0.001217	0.997396 ± 0.000381	0.998010 ± 0.000481
ΔR^2^ (Train—Validation gap)	0.000014	0.000235	0.000176	0.000191
ΔR^2^ (Train—Test gap)	0.000075	0.00239	0.000326	0.00067
MAE ratio (Validation/Train)	1.023778	1.084707	1.030415	0.991422
MAE ratio (Test/Train)	1.051461	1.141466	1.021729	1.105296
RMSE ratio (Validation/Train)	1.009129	1.063041	1.015116	0.992686
RMSE ratio (Test/Train)	1.058062	1.383225	1.034069	1.223264

**Table 3:** Torque–rotation value of four models from numerical simulation, experiment, and ANN.

	Model 1			Model 2			Model 3			Model 4		
	CAE	EXP	ANN	CAE	EXP	ANN	CAE	Exp	ANN	CAE	EXP	ANN
Constant-torque angle (°)	23–75	23–125	25–125	16–43	15–46	17–45	22–51	20–57	20–60	23–44	20–45	20–46
Constant torque (N·m)	0.165	0.124	0.127	0.139	0.174	0.172	0.128	0.136	0.139	0.142	0.167	0.164

## Data Availability

The original contributions presented in this study are included in the article. Further inquiries can be directed to the corresponding author.
